# A novel technique for negotiation of a complex fistula-in-ano using a flexible ureteral catheter

**DOI:** 10.1308/rcsann.2014.96.1.80

**Published:** 2014-01

**Authors:** NC Tanner, A Maw

**Affiliations:** Betsi Cadwaladr University Health Board,UK

## BACKGROUND

Insertion of a seton into a complex fistula-in-ano can be challenging due to the variable course of the track to reach the internal opening. If the internal opening is identified easily, then passage of a seton is a useful initial step in management. Commonly, negotiation of the track cannot be achieved easily, even when using a malleable probe.

## TECHNIQUE

**Figure 1:**
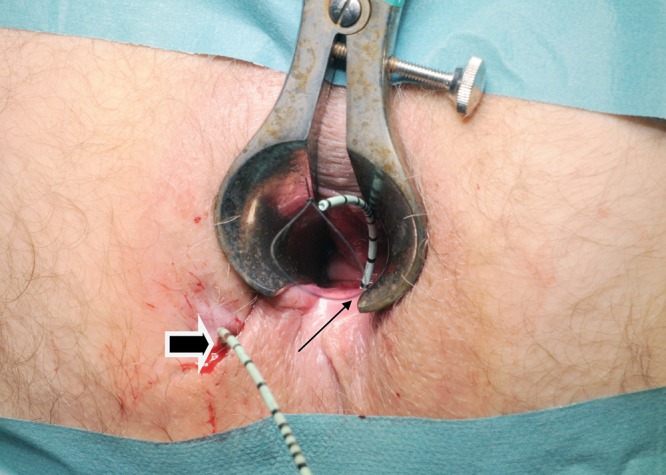
Ureteral catheter and nylon suture inserted through a complex fistula-in-ano (thick arrow = external opening; thin arrow = internal opening)

**Figure 2:**
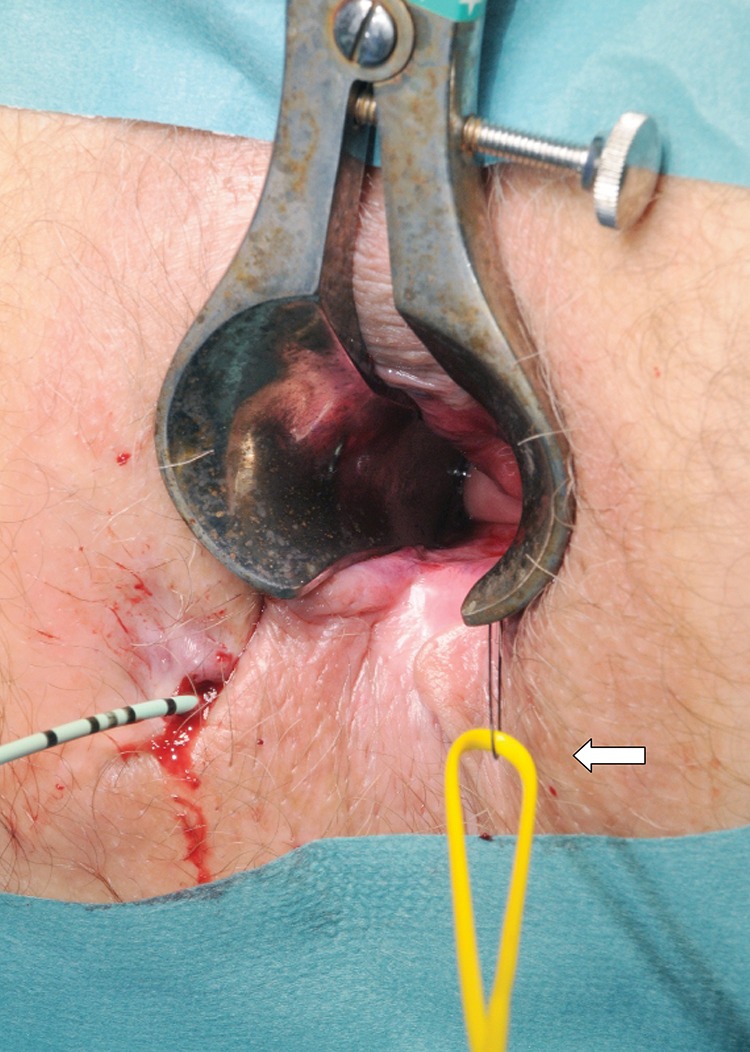
Passage of a vascular sling (arrow) through the fistula track

**Figure 3:**
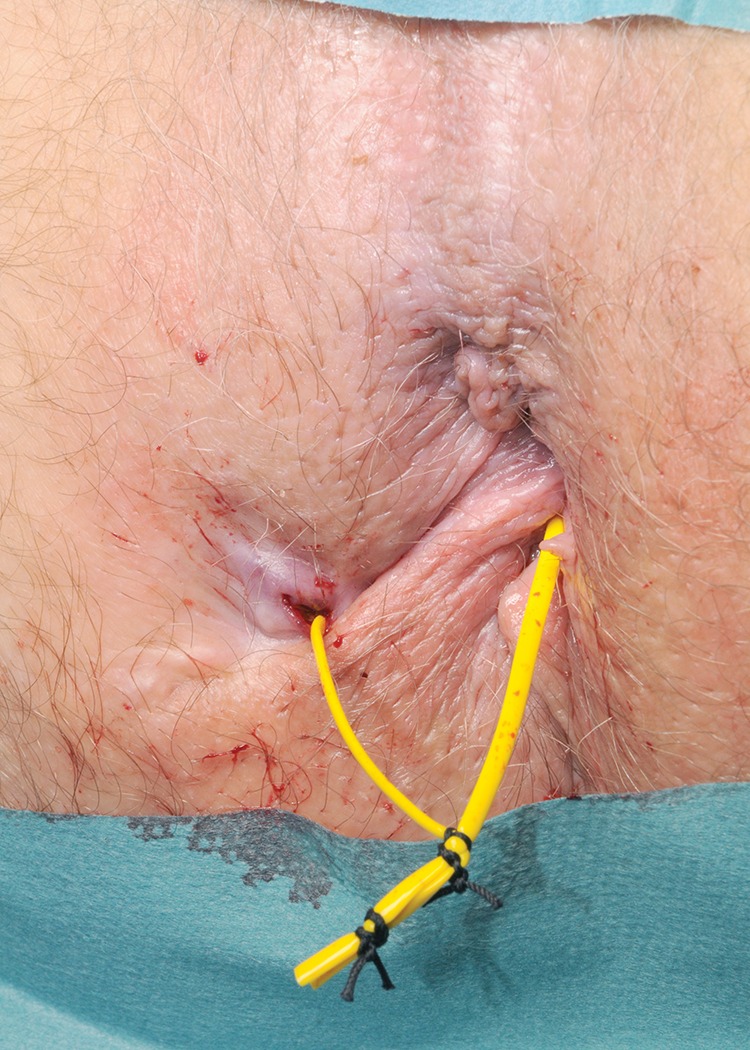
Loose seton in situ

On examination, the external opening is identified and hydrogen peroxide instillation is used to detect an internal opening. The flexible end of a 0.89mm diameter urological guidewire coated in polytetrafluoroethylene (Boston Scientific, Natick, MA, US) is inserted through the internal opening. Using manual palpation and artery forceps, the guidewire is advanced until it protrudes beyond the external opening. Following this, a 6Fr open-end ureteral catheter (Cook Medical, Bloomington, IN, US) is advanced over the guidewire. A 1/0 nylon suture is passed through the lumen of the catheter and a loop is tied in the distal end of the suture (Fig 1), allowing a vascular sling to be pulled through the track, from the internal to the external opening (Fig 2). This is then fixed to form a loose seton (Fig 3).

## DISCUSSION

Seton insertion is common practice for fistula-in-ano management to allow drainage of infective material, preventing abscess formation. The posterior fistula track is rarely straight so forced insertion of an inflexible probe can create false passages. This technique allows the flexible wire to negotiate the tortuous track easily without this risk and has been used successfully in one patient.

